# Nasal Branch of the Anterior Ethmoidal Artery and Cribroethmoidal Groove: New Frontal Sinus Landmarks

**DOI:** 10.1002/lary.70041

**Published:** 2025-08-13

**Authors:** Alessandro Vinciguerra, Mario Turri‐Zanoni, Pierfrancesco Bettini, Alberto Daniele Arosio, Alberto Gandolfi, Daniel Balzi, Gloria Schiavo, Federico Russo, Marco Valentini, Marco Ferrari, Paolo Castelnuovo, Philippe Herman, Maurizio Bignami, Paolo Battaglia

**Affiliations:** ^1^ Division of Otorhinolaryngology, Department of Biotechnology and Life Sciences University of Insubria, ASST Lariana Como Italy; ^2^ ENT and Audiology Department University of Ferrara Ferrara Italy; ^3^ Division of Otorhinolaryngology, Department of Biotechnology and Life Sciences University of Insubria, Ospedale di Circolo e Fondazione Macchi Varese Italy; ^4^ Section of Otorhinolaryngology—Head and Neck Surgery, Department of Neuroscience University of Padova Padova Italy; ^5^ Otorhinolaryngology and Skull Base Center AP‐HP, Hospital Lariboisière Paris France

**Keywords:** anterior ethmoidal artery, endoscopic sinus surgery, epistaxis, frontal sinus, skull base surgery

## Abstract

**Objective:**

Expanded frontal sinusotomies (Draf IIb/c‐III) are essential surgical procedures for managing complex frontal sinus pathologies. However, traditional anatomical landmarks may be difficult to identify, particularly in tumor or revision cases. This manuscript investigates the nasal branch of the anterior ethmoidal artery (NbAEA) and the cribroethmoidal groove (CrEGr) as reliable and consistent landmarks for endoscopic frontal sinusotomies.

**Methods:**

This study included anatomical dissections (medio‐lateral approach) on three fresh cadavers (six sides) focused on the region anterior to the first olfactory phylum, namely the cribo‐frontal area. Additionally, a retrospective clinical case series of patients that underwent centripetal dissection with a medio‐lateral approach to the frontal sinus was performed. Identification of NbAEA, CrEGr, and the first olfactory phylum, along with surgical outcomes and complications, was analyzed.

**Results:**

The NbAEA and CrEGr were identified in all dissected sides and were located anterior to the first olfactory phylum. Considering the 19 enrolled patients, 13/19 (68.4%) were treated with a bilateral centripetal dissection and Draf III procedure (26 sides); whereas 6/19 patients (31.6%) underwent a unilateral approach with a Draf IIb/c procedure. The NbAEA and CrEGr were identified in all cases (100%) and in only 9/19 cases (47.7%) the first olfactory phylum was additionally exposed, reinforcing the role of these new anatomical landmarks. No perioperative complications were recorded.

**Conclusions:**

This study supports the clinical significance of NbAEA and CrEGr as reliable anatomical landmarks, and their identification in 100% of cases reinforces their practical applicability in surgical approaches to the frontal sinus.

**Level of Evidence:**

Level 4.

## Graphical abstract

This study investigates the nasal branch of the anterior ethmoidal artery (NbAEA) and the cribroethmoidal groove (CrEGr) as novel and reliable anatomical landmarks for expanded endoscopic frontal sinusotomies. Through cadaveric dissections and a clinical case series, both structures were consistently identified and shown to aid safe and accurate surgical access to the frontal sinus. These findings support their clinical applicability, especially in challenging or revision cases.

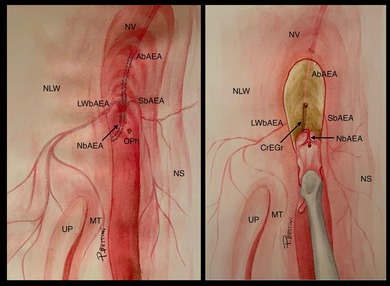

## 1 Introduction

Endoscopic approach to the frontal sinus (FS) is a pivotal procedure in endoscopic endonasal surgery, both in functional and expanded approaches, with significant risk of complications (i.e., CSF leak, iatrogenic stenosis) due to the variable anatomy among individuals [1]. Over the last decades, multiple anatomical landmarks have been described to facilitate the intraoperative FS identification, thus enabling subsequent frontal sinusotomy. Nevertheless, if “functional” frontal sinusotomies, such as Draf I‐IIa, depend on individual anatomy and present the surgical concept of maximal functional outcome with the least aggressive surgical procedure, the principle is different for expanded endonasal FS approaches (Draf IIb/c‐III) (Table 1) [2, 3]. Indeed, the latter procedures are generally applied in case of distorted anatomy (i.e., scar tissue, revision surgery, type 2 inflammation, tumor) and are based on the surgical principle of achieving the maximal anatomical result, both in a unilateral (Draf IIb/c) or bilateral approach (Draf III) and are classically guided by renowned anatomical landmarks [4, 5, 6]. Among all, it should be cited the axilla of the middle turbinate, the lamina papyracea, the lacrimal sac, the first olfactory phylum, and the nasal bones [1]. However, in clinical practice, it is often difficult to accurately identify these anatomical landmarks, making the FS expanded approaches often disorienting.

**TABLE 1:** Classification of endoscopic approaches to the frontal sinus.

Procedure	Draf classification	Description
Functional	Draf I	Anterior ethmoidectomy without touching the frontal sinus outflow pathway
	Draf IIa	Removal of the anterior ethmoidal cells creating an opening between the middle turbinate and the lamina papyracea
Expanded	Draf IIb	Removal of the frontal sinus floor between the nasal septum and the lamina papyracea
	Draf IIc	Draf IIb associated with removal of the interfrontal sinus septum
	Draf III	Bilateral removal of the floor of the frontal sinus anterior to the middle turbinates from one lamina papyracea to the next with superior septectomy and inter sinus septectomy

In the last few years, the new understanding of the nasal branch of the anterior ethmoidal artery (NbAEA) and related anatomy has paved the way for new anatomical landmarks that could potentially be of interest in FS surgery [7]; however, to date, this consideration has remained only anecdotical and without a real clinical application [8].

### 1.1 Anatomy of the Anterior Ethmoidal Artery

**FIGURE 1:**
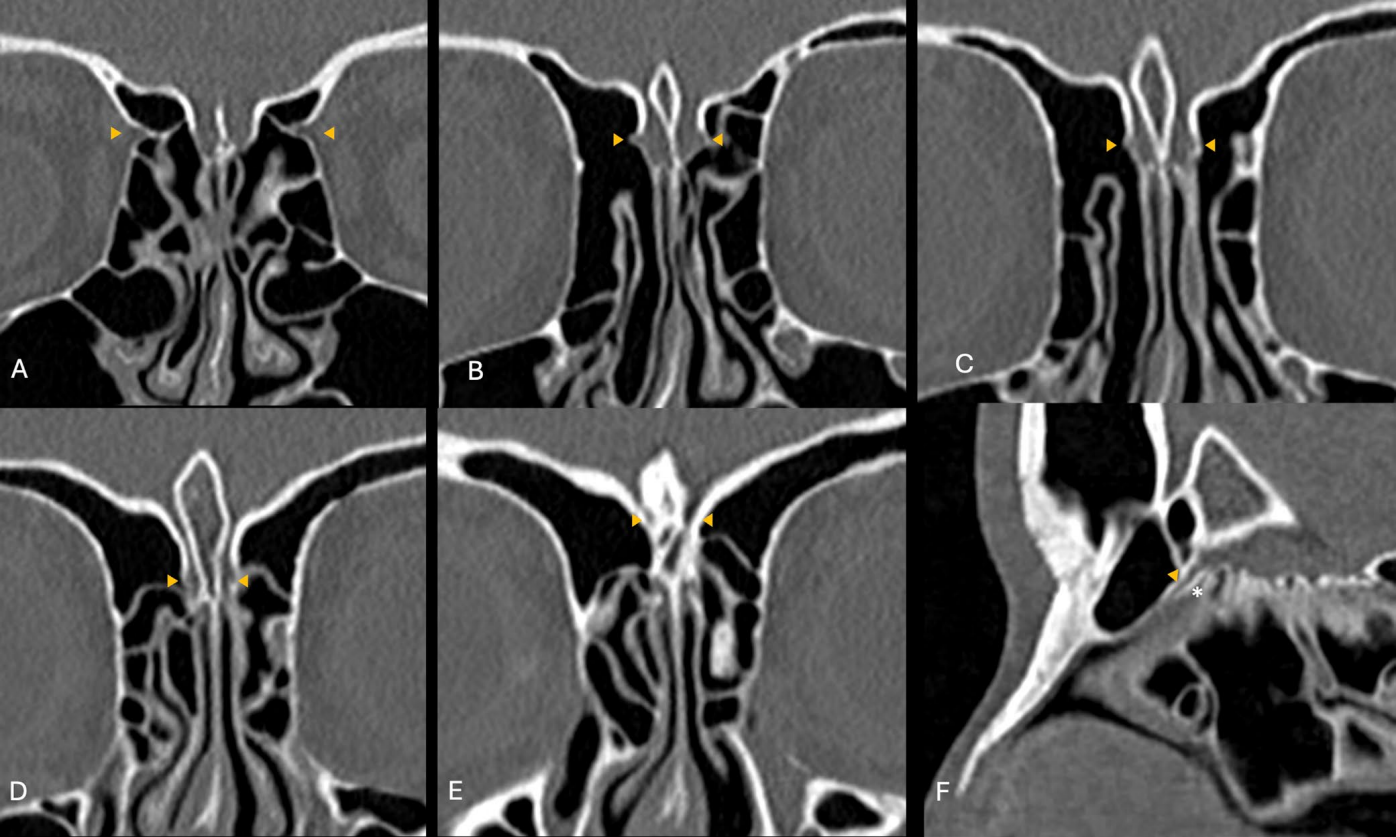
Radiologic analysis of the AEA on CT scans in coronal (A–E) and sagittal (F) planes. Once the AEA enters into the cranial fossa it gives two terminal branches: The anterior meningeal artery and nasal branch. Yellow arrows show the AEA (A–E) and NbAEA (F).

**FIGURE 2:**
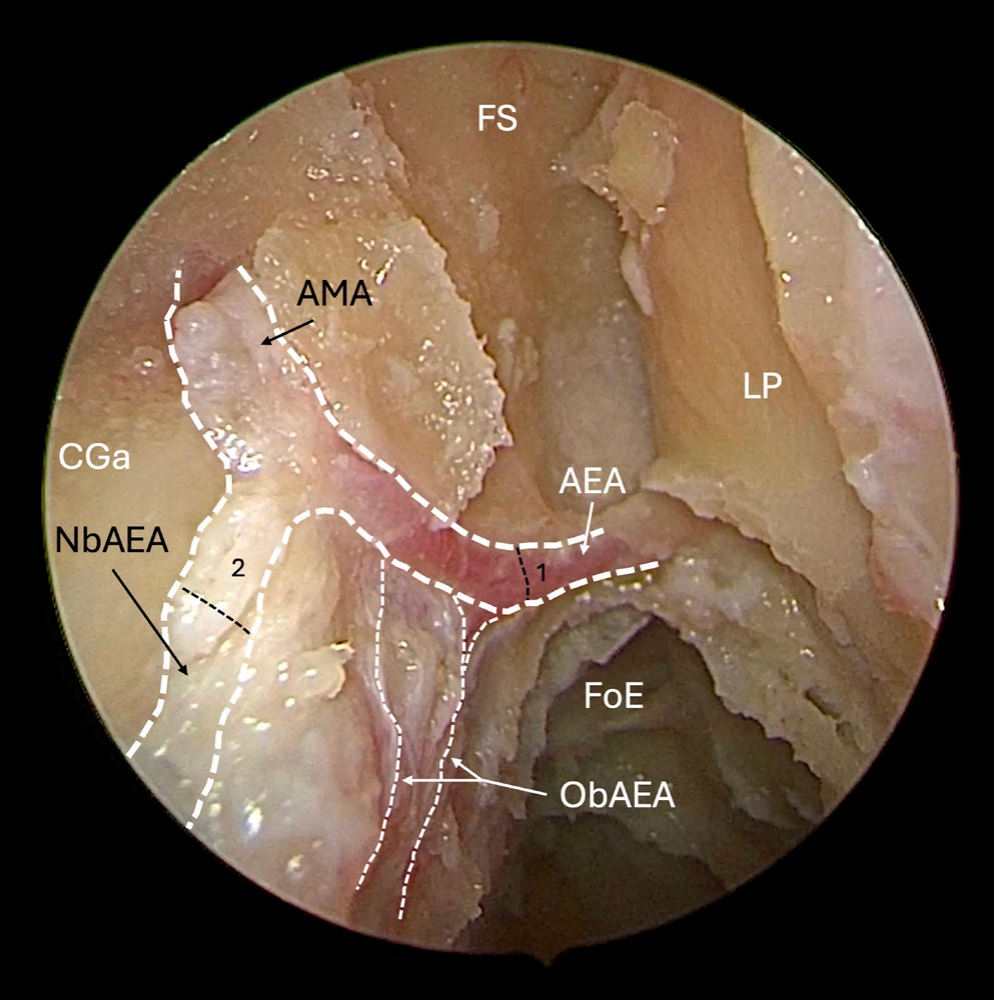
Anatomical study of the left cribriform area after having performed a Draf III procedure and removed the lateral lamella. 1, vertical dotted line etmoidal sulcus, ethmoidal sulcus; 2, dotted line, cribroethmoidal foramen; AEA, anterior ethmoidal artery; AMA, anterior meningeal artery; CGA, crista galli of the ethmoid bone; CrEGr, cribroethmoidal groove; FS, frontal sinus; FoE, fovea ethmoidalis; LP, lamina papiracea; NbAEA, nasal branch of the anterior ethmoidal artery; ObAEA, olfactory branch of the AEA. [Color figure can be viewed in the online issue, which is available at www.laryngoscope.com]

The AEA leaves the orbital cavity through the anterior ethmoidal foramen, traverses the ethmoidal roof via the ethmoidal canal (with varying degrees of dehiscence), and enters the anterior cranial fossa through the lateral lamella at the level of the ethmoidal sulcus (bony sulcus on the lateral walls of the olfactory fossae) (Figure 1) [9, 10]. Endocranially, the AEA runs in an epidural plane, called the anterior ethmoidal groove, and gives off inferior branches that supply the anterior cribriform plate. Its main trunk continues anteriorly and divides into two branches: one gives rise to an anterior meningeal artery (AMA), while the other, also called NbAEA, crosses the olfactory bulb and enters inside the nasal fossa through the cribroethmoidal foramen, a few millimeters anterior to the first olfactory phylum (Figure 2). Inside the nasal cavity, the NbAEA runs anteriorly within a bony sulcus, the so‐called cribroethmoidal groove (CrEGr), which is located at the level of the endonasal projection of the FS floor, namely the cribo‐frontal area [11, 12]. During its pathway, it gives off three main branches: an external branch, which supplies the anterolateral wall of the nasal cavity (LWbAEA, lateral wall branch of the AEA), a medial branch, which supplies the anterior portion of the nasal septum (SbAEA, septal branch of the AEA), and an anterior branch (AbAEA, anterior nasal branch), which represents the natural anterior prosecution of the NbAEA. The latter then passes inside a canal in the width of nasal bones, the cribroethmoidal canal, and continues anteriorly to supply, thanks to the anastomosis with the facial artery, the most anterior portion of the nasal roof [13, 14, 15].

As a result of the abovementioned considerations, two main surgical implications should be underlined: (1) the NbAEA represents the main AEA branch to the nasal fossa, entering this cavity a few millimeters anteriorly to the first olfactory phylum; (2) once inside the nasal fossa, it runs in a bony sulcus (CrEGr), where it gives off three terminal branches (AbAEA, SbAEA, and LWbAEA).

This manuscript aims to investigate the NbAEA and CrEGr, aiming to provide reliable and consistent landmarks for endoscopic expanded frontal sinusotomies.

## 2 Methods

### 2.1 Anatomical Dissection Study

The preliminary anatomical study was conducted on three fresh cadaveric specimens (six sides) that were prepared with intravascular injection of colored silicone. The surgical dissection was performed by three surgeons (A.V., M.T.Z, and P.B.) using paranasal sinus and skull base endoscopic instruments (Karl Storz, Tuttlingen, Germany), high‐speed drill with angled handpiece and diamond cutting burrs (Karl Storz), and 0°–45° endoscope coupled to a high‐definition camera and monitor (Karl Storz Endoscopy). The dissection process was focused on the anatomy of the region anterior to the first olfactory phylum, namely the endonasal projection of the FS floor.

### 2.2 Clinical Case‐Series

**FIGURE 3:**
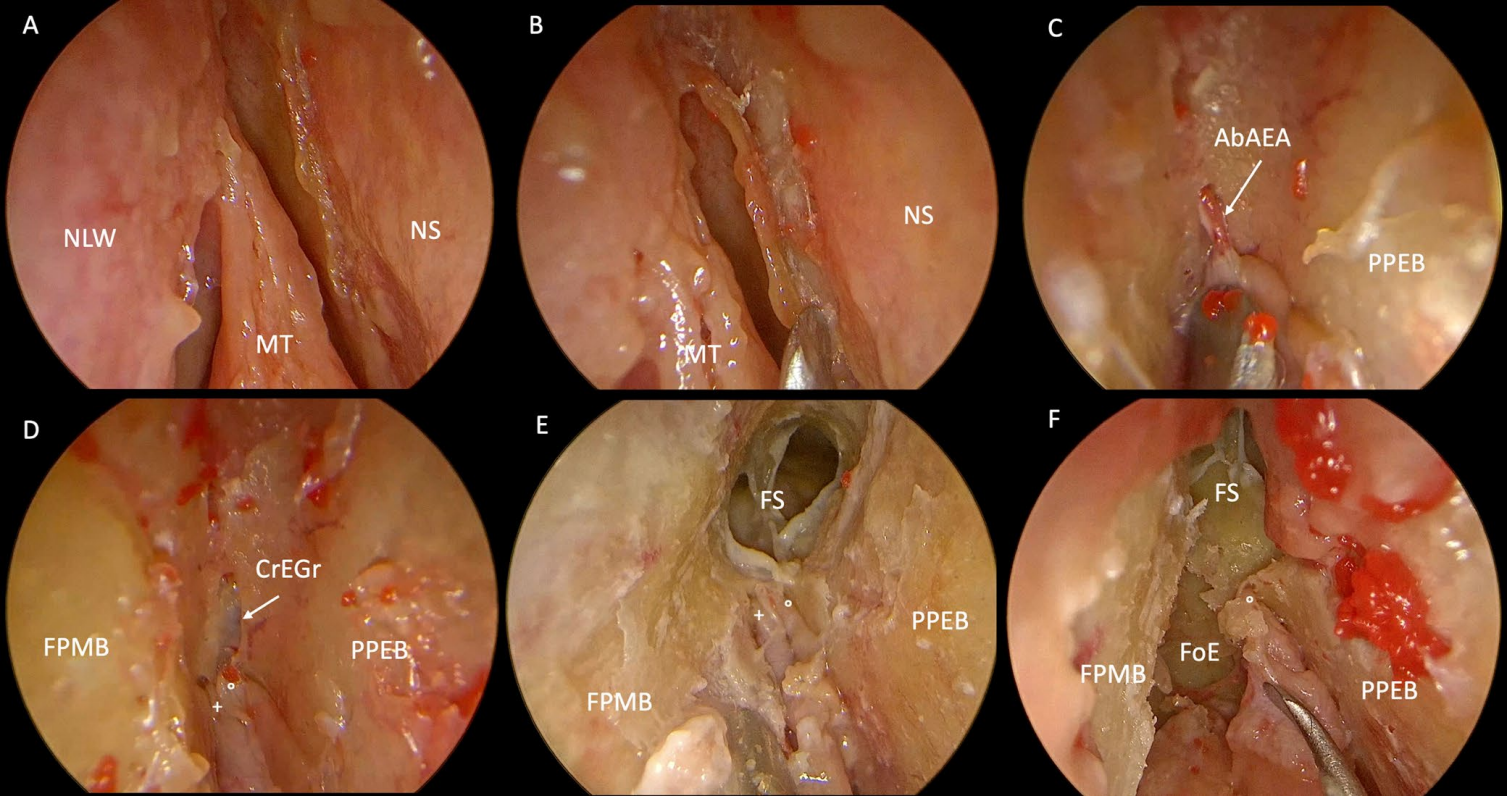
Anatomical study of a right frontal sinusotomy type Draf IIb performed in a medio‐lateral approach. °, NbAEA; +, first olfactory phylum; AbAEA, anterior branch of the anterior ethmoidal artery; CrEGr, cribroethmoidal groove; FoE, fovea ethmoidalis; FPMB, frontal process of the maxillary bone; FS, frontal sinus; MT, middle turbinate; NLW, lateral nasal wall; NS, nasal septum; PPEB, perpendicular process of the ethmoidal bone. [Color figure can be viewed in the online issue, which is available at www.laryngoscope.com]

**FIGURE 4:**
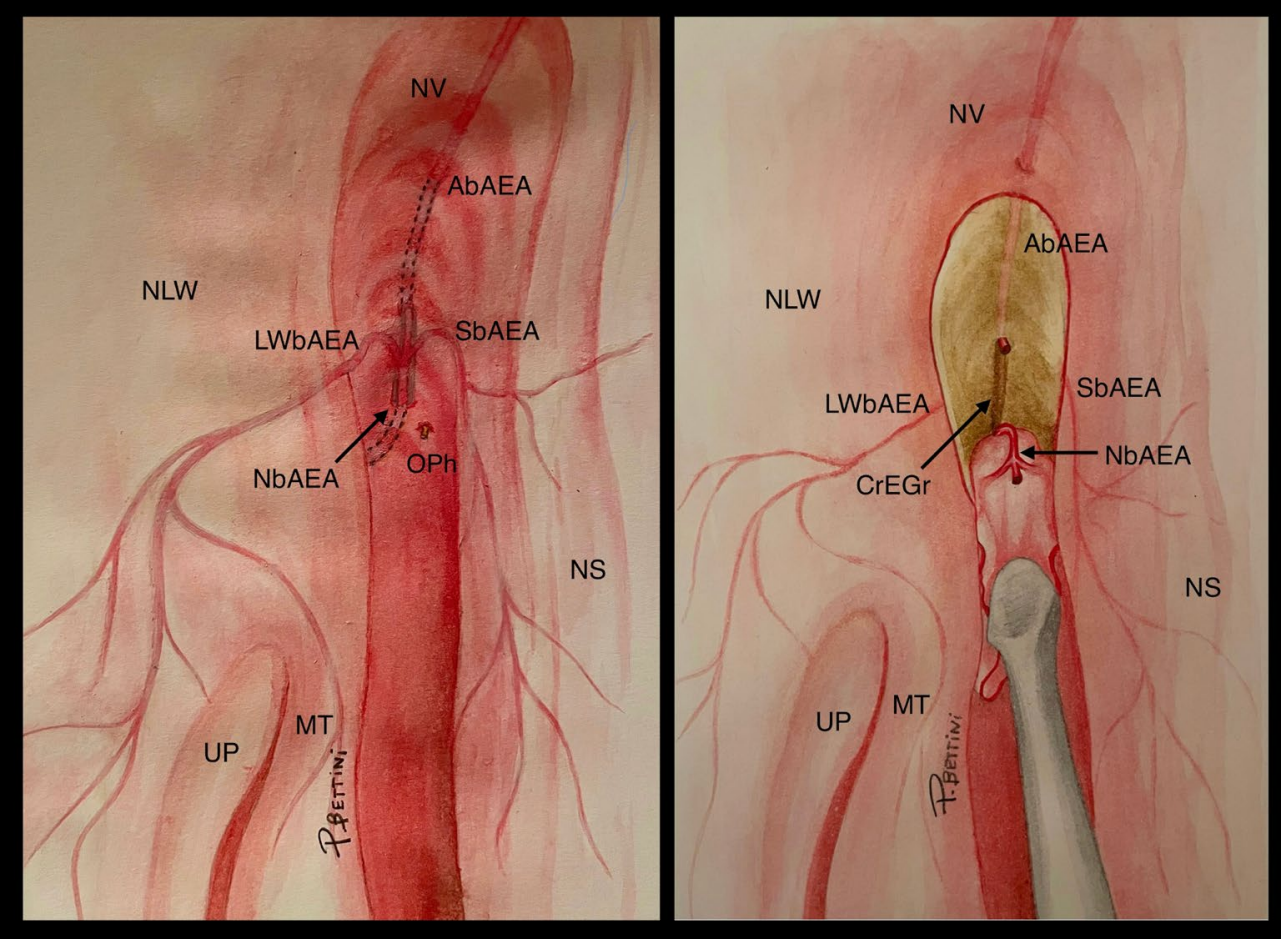
Graphical representation of the endoscopic view of a right nasal fossa with the AEA nasal branches. AbAEA, anterior branch of the AEA; CrEGr, cribroethmoidal groove; LWbAEA, lateral wall branch of the AEA; MT, middle turbinate; NbAEA, nasal branch of the AEA; NLW, nasal lateral wall; NS, nasal septum; NV, nasal vault; OPh, olfactory phylum; SbAEA, septal branch of the AEA; UP, uncinate process. [Color figure can be viewed in the online issue, which is available at www.laryngoscope.com]

**FIGURE 5:**
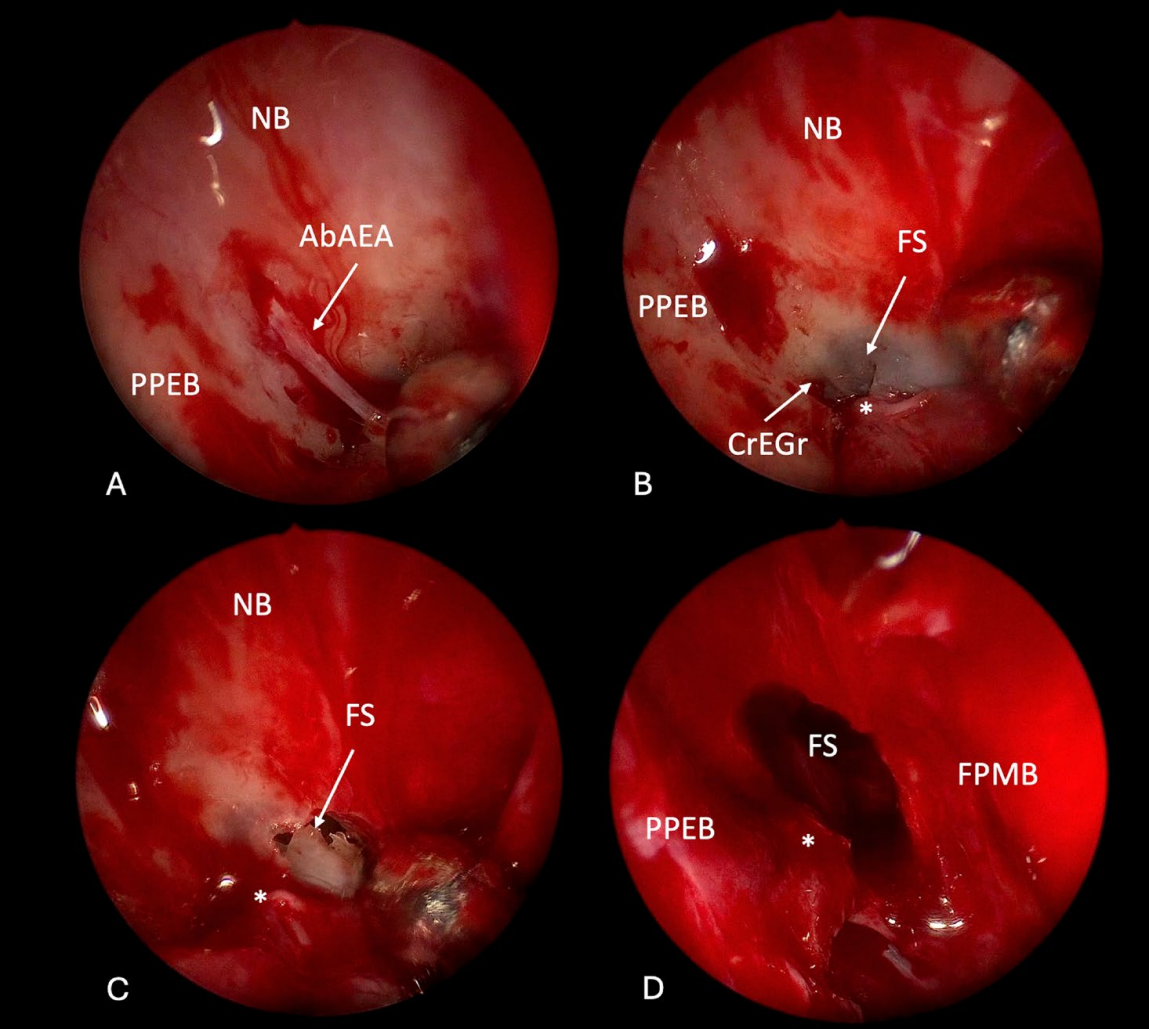
Intraoperative view of a left Draf IIb performed for an iatrogenic stenosis of the frontal sinus. AbAEA, anterior branch of the anterior ethmoidal artery; CrEGr, cribroetmoidal groove; FPMB, frontal process of the maxillary bone; FS, frontal sinus; NB, nasal bones; NS, nasal septum; PPEB, perpendicular process of the ethmoidal bone. [Color figure can be viewed in the online issue, which is available at www.laryngoscope.com]

A consecutive series of patients operated with a centripetal dissection and an outside‐in approach to the FS to treat malignancies of the sinonasal cavity were retrospectively included. Under general anesthesia and using a 0°–45° endoscope, a “U” shape incision was made from the head of the middle turbinate to the nasal vault with an additional release incision on the septum (Figure 3). Subperiosteal dissection follows, exposing the AbAEA, which is then cut. Following the subperiosteal plane, the CrEGr is exposed to its posterior portion, where the origin of the NbAEA is identified, just a few millimeters anterior to the first olfactory phylum (Figure 4). Drilling then started at the level of the CrEGr, taking care to stay anterior to its proximal portion that can also be identified with the NbAEA origin. Once the FS is opened, it can be enlarged medio‐laterally and then anteriorly. At the end of the procedure, the posterior‐based flap can be repositioned to cover the posterior border of the frontal sinusotomy (Draf IIb/III) (Figure 5).

Among all surgical procedures, the following information was recorded: identification and section of the AbAEA, presence of the CrEGr, and identification of the first olfactory phylum. In addition to this, data concerning age, sex, type of Draf procedure performed, pathology treated, and peri‐operatory complications (namely lesion of the first olfactory phylum and postoperative epistaxis) were also recorded. Data are reported as median, IQ range, and/or percentage.

Patients were recruited between June 2024 and January 2025 and treated in the two hospitals of Università dell'Insubria, Italy (ASST Lariana Como and Ospedale di Circolo e Fondazione Macchi, Varese). The study was conducted according to the ethical standards established in the 1964 Declaration of Helsinki and revised in 2011 and was approved by the Ethics at Insubria (approval number 0033025/2015). Informed consent was obtained from all patients, and the study was conducted according to the ethical standards of the Declaration of Helsinki revised in 2011.

Follow‐up visits were scheduled at 10–30 days, recording peri‐operative complications occurrence; subsequent ENT examinations were based on the treated pathology.

The primary objective of this study was to define the reliability and safety of the NbAEA and CrEGr as anatomical landmarks to identify the FS in a medio‐lateral approach.

## 3 Results

### 3.1 Anatomical Study

In all dissected specimens, the AbAEA was directly viewed and cut, exposing in 100% of cases the CrEGr which guides the surgeon to the origin of the NbAEA, located just anteriorly to the first olfactory phylum. The CrEGr median length was 4.2 mm (range 2.8–6.5 mm); the first olfactory phylum was identified and visualized in all cases posterior to the NbAEA.

In the six dissected sides, the FS was opened by drilling at the level of its floor, staying anterior to the NbAEA origin, specifically at the level of CrEGr.

### 3.2 Clinical Case Series

The median age of the 19 enrolled patients was 66 years (IQ range 55–77), with a male to female ratio of 14:5.13/19 (68.4%) cases were treated with a bilateral centripetal dissection and Draf III procedure to gain access to the FS (26 sides); whereas 6/19 patients (31.6%) underwent a unilateral approach and Draf IIb‐c procedure (Table 2).

**TABLE 2:** Main clinical characteristics.

Patients number	19
Age, median (range)	66 (55–77)
Gender, *N* (%)	
Male	14 (73.7)
Female	5 (26.3)
Histology treated, *N* (%)	
(n)ITAC	8 (42.1)
ONB	4 (21)
SCC	2 (10.5)
Melanoma	2 (10.5)
INI‐1 deficient	1 (5.3)
Meningioma	1 (5.3)
Glomagioperycitoma	1 (5.3)
TNM staging (ethmoid sinus tumors), *N* (%)	
T3	13 (68.5)
T4a	4 (21)
None	2 (10.5)
Side of the pathology, *N* (%)	
Left	8 (42.1)
Right	6 (31.6)
Bilateral	5 (26.3)
Treated side, *N* (%)	
Bilateral	13 (68.4)
Unilateral	6 (31.6)
Draf procedure performed, *N*° (%)	
Draf III	13 (68.4)
Draf IIb	6 (31.6)

*Note*: TNM staging was stated accordingly to the 8th edition.

Abbreviations: (n)ITAC, (non)intestinal type adenocarcinoma; ONB, olfactory neuroblastoma; SCC, squamous cell carcinoma.

The AbAEA was always identified and sectioned, exposing the CrEGr in 100% of cases either treated with a unilateral approach (31.6%) or a bilateral approach (68.4%), both on the “pathologic” and contralateral side. In 9/19 cases (47.7%) the first olfactory phylum was additionally exposed, whereas in 10/19 cases (52.3%), the exposure of this posterior landmark was considered unnecessary. The NbAEA was identified at its origin (cribroethmoidal foramen) in all cases (100%).

No perioperative complications were recorded; specifically considering bleeding episodes and unintentional CSF leaks. All clinical characteristics are shown in Table 1.

## 4 Discussion

The main finding of our manuscript is that NbAEA and CrEGr represent reliable and consistent landmarks for endoscopic expanded frontal sinusotomies (Draf IIb/c‐III), being identified in 100% of cases both in the preclinical setting and in vivo surgeries. To the best of our knowledge, this study is the first that explores the practical applicability of these anatomical structures in the surgical approach to the FS.

Over the last two decades, expanded endoscopic frontal sinusotomies have gained ground on more “traditional” external routes, such as the osteoplastic flap approach, for addressing most recalcitrant benign chronic inflammatory disease or neoplasms of the FS given the reduced morbidity and high success rate [5, 16, 17]. From a technical point of view, these endoscopic approaches (Draf IIb/c‐III) can be performed in two different ways: the latero‐medial or medio‐lateral approach, also known, in relation to the ethmoidal corridor, as inside‐out or outside‐in techniques, respectively [18].

The latter has been proposed to be the most reliable approach when the frontal recess anatomy is difficult, and sometimes impossible to define because of scar tissue or pathology [17, 19]. Indeed, the outside‐in technique presents codified anatomical landmarks that help the surgeon in identifying safely the FS: posteriorly, the first olfactory phylum; laterally, the medial orbital wall and lacrimal pathway; anteriorly, the nasal bones [19]. As a result, the pivotal concept of the outside‐in technique of expanded frontal sinusotomies is that the dissection is not related to frontal recess anatomy, and the FS opening is always obtained anteriorly to the first olfactory phylum, which demarks the anterior limit of the anterior cranial fossa [20]. Nevertheless, in a clinical setting, this landmark can be difficult to identify, exposing the patient to potential CSF leak, particularly in unexperienced hands [17, 21]. In the iconographic descriptions of this surgical approach, many authors state that before the first olfactory phylum, a “sentinel” vascular structure, known as emissary veins, is encountered and potentially alerts the surgeon to be close to the olfactory cleft [17, 19]. In 2020, Roussel et al. [10] thoroughly explored the region anterior to the first olfactory phylum, leading to the revised anatomical concept of the NbAEA (Figures 3 and 4). Specifically, the AEA enters inside the nasal fossa with the NbAEA anteriorly to the first olfactory phylum and then runs anteriorly in a bony sulcus, known as CrEGr, at which level it gives its three terminal branches (AbAEA, SbAEA, and LWbAEA) [11].

When applying these anatomical concepts to the outside‐in expanded frontal sinusotomies, different considerations arise. During the subperiosteal dissection to expose the cribo‐frontal area, the only vessel present is the NbAEA and its branches, which most likely represent the so‐called “emissary veins” described previously particularly, the first vascular structure encountered is its anterior branch (AbAEA) which must be cut to follow the subperiosteal dissection, exposing the CrEGr (Figure 4) [10]. This bony sulcus is a constant landmark that, in our preclinical series, measures 4.2 mm (range 2.8–6.5 mm), slightly longer compared to previous data (2.43 mm, range 0–7 mm), in which the NbAEA passes [11]. The CrEGr is a new landmark in FS surgery and is useful for several reasons. First, it guides the surgeon to the NbAEA origin and indirectly to the first olfactory phylum: indeed, as already demonstrated, the NbAEA enters inside the nasal fossa 2.86 ± 1.93 mm (range, 1–7 mm) anteriorly to the first olfactory phylum [11]. By following the CrEGr, the posterior limit of the frontal sinusotomy can be safely identified at the proximal (i.e., posterior) NbAEA end, practically attached to the first olfactory phylum. This aspect is confirmed by our clinical series where, in 52.3% of cases, the NbAEA was considered sufficient to identify the posterior limit of the expanded frontal sinusotomies, while the exposition of the first olfactory phylum was additionally performed in only 47.7% of the cases.

Second, pointing to the NbAEA origin, dissection of the CrEGr prevents any devascularization of the posterior based flap [22]. Indeed, following expanded frontal sinusotomies, the most relevant complication is neo‐ostium stenosis which may occur up to 2 years after the surgery and is primarily caused by uncovered drilled bone [1]. To prevent this complication, different flaps have been described to minimize the area of exposed bone, but in the majority of cases, the risk of flaps devascularization is significant. By identifying the CrEGr, the pedicle of the posterior based flap can be identified and preserved, reinforcing the role of this flap with a solid vascular pedicle, in the resurfacing after expanded frontal sinusotomies [7, 23]. Moreover, as recently demonstrated, keeping this bony sulcus as a guide to the NbAEA origin helps manage the AEA‐based epistaxis with no cauterization/ligation of the anterior ethmoidal artery at the level of the ethmoidal roof, which potentially exposes the patient to complications [9, 24]. As a result, the CrEGr can be used both to preserve the vascularization of the posterior based flap and to safely address AEA‐based epistaxis [9].

Third, considering that the CrEGr lies just anterior to the first olfactory phylum, it represents a bony landmark that indicates to the surgeon exactly where to start drilling since this bony cleft is located at the endonasal projection of the FS floor [1]. Indeed, even if a number of landmarks can guide the surgeon to imagine where to start the drilling, several authors underlined the importance of stereotactic image guidance system in this FS approach to exactly identify where to open the FS [17]. Nevertheless, the additional identification of a bony sulcus that delimits the area of drilling (generally with curved instruments, 30°–45° drills) may facilitate the surgeon to enter safely inside the FS and then enlarge the sinusotomies keeping the posterior and anterior plate of the FS as intrafrontal landmarks to maximize the sinus opening.

Some may argue that these structures may be challenging to identify; however, as demonstrated in our preclinical setting and in vivo series, the NbAEA and CrEGr are consistent and reliable structures in 100% of cases, thus adding new surgical landmarks to the armamentarium of a skull base surgeon. Nevertheless, these structures should be considered complementary landmarks rather than exclusive ones, especially for surgeons with less experience in advanced FS surgery. Indeed, the NbAEA and CrEGr offer valuable orientation in cases of distorted anatomy or in revision procedures where traditional landmarks may be unreliable or absent, by adding additional structures to guide the surgeon safely to the FS.

Despite its strengths, this study has some limitations. The relatively small sample size and retrospective nature of the clinical analysis may introduce biases in data interpretation. Additionally, the absence of a control group limits the direct comparison with traditional anatomical landmarks. Further prospective studies with larger cohorts, longer follow‐up, and intraoperative navigation would help validate these findings and refine their clinical utility in FS surgery.

## 5 Conclusion

Our study supports the clinical significance of NbAEA and CrEGr as reliable and consistent anatomical landmarks for expanded endoscopic frontal sinusotomies. Their identification in 100% of cases reinforces their practical applicability in surgical approaches to the FS. The CrEGr serves as a crucial bony guide, enhancing surgical precision and minimizing complications. These findings provide a refined anatomical framework that can improve surgical outcomes, particularly for complex FS cases. Further studies and clinical validations will help integrate these concepts into routine surgical practice, optimizing patient safety and procedural efficiency in FS surgery.

## Disclosure

The authors have nothing to report.

## Conflicts of Interest

The authors declare no conflicts of interest.

## Data Availability

The data that support the findings of this study are available on request from the corresponding author. The data are not publicly available due to privacy or ethical restrictions.
